# Supplementary material for: Insights into dog owner perspectives on risks, benefits, and nutritional value of raw diets compared to commercial cooked diets
Source: PeerJ. 2020 Dec 8;8:e10383. doi: 10.7717/peerj.10383 (PMC7731655; doi:10.7717/peerj.10383)
Supplement: Supplemental Information 1 [file peerj-08-10383-s001.pdf]

Dog Owner Perceptions

Hello! Our names are Alysia Empert-Gallegos and Sally Poole and we are veterinary students at the University of Glasgow in the United Kingdom. We would like to invite you to take our questionnaire. It will take you approximately 10-15 minutes.

We are undertaking a research project looking into dog owner perceptions about canine feeding and dog food. There are no known risks associated with this questionnaire. Taking part in this study is completely voluntary, and you may terminate your participation and exit the survey at any time, without penalty. You may skip any questions you do not wish to answer. Your participation in this research will be completely confidential, and data will be analyzed anonymously.

By consenting to complete this survey, you agree with the above terms, are over 18 years of age and are the primary caregiver or owner to at least one pet dog.

For further information please contact Philippa.Yam@glasgow.ac.uk

Thank you for participating in this study!

1. **Do you consent to the above terms**

Mark only one oval.

- ⬭ Yes
- ⬭ No *Skip to "Thank you for completing this survey!."*

About You

2. **In what country do you currently reside?**
Mark only one oval.

- Afghanistan
- Akrotiri
- Albania
- Algeria
- American Samoa
- Andorra
- Angola
- Anguilla
- Antarctica
- Antigua and Barbuda
- Argentina
- Armenia
- Aruba
- Ashmore and Cartier Islands
- Australia
- Austria
- Azerbaijan
- Bahamas, The
- Bahrain
- Bangladesh
- Barbados
- Bassas da India
- Belarus
- Belgium
- Belize
- Benin
- Bermuda
- Bhutan
- Bolivia
- Bosnia and Herzegovina
- Botswana
- Bouvet Island
- Brazil
- British Indian Ocean Territory
- British Virgin Islands
- Brunei
- Bulgaria
- Burkina Faso
- Burma
- Burundi
- Cambodia

- Cameroon
- Canada
- Cape Verde
- Cayman Islands
- Central African Republic
- Chad
- Chile
- China
- Christmas Island
- Clipperton Island
- Cocos (Keeling) Islands
- Colombia
- Comoros
- Congo, Democratic Republic of the
- Congo, Republic of the
- Cook Islands
- Coral Sea Islands
- Costa Rica
- Cote d'Ivoire
- Croatia
- Cuba
- Cyprus
- Czech Republic
- Denmark
- Dhekelia
- Djibouti
- Dominica
- Dominican Republic
- Ecuador
- Egypt
- El Salvador
- Equatorial Guinea
- Eritrea
- Estonia
- Ethiopia
- Europa Island
- Falkland Islands (Islas Malvinas)
- Faroe Islands
- Fiji
- Finland
- France
- French Guiana

- French Polynesia
- French Southern and Antarctic Lands
- Gabon
- Gambia, The
- Gaza Strip
- Georgia
- Germany
- Ghana
- Gibraltar
- Glorioso Islands
- Greece
- Greenland
- Grenada
- Guadeloupe
- Guam
- Guatemala
- Guernsey
- Guinea
- Guinea-Bissau
- Guyana
- Haiti
- Heard Island and McDonald Islands
- Holy See (Vatican City)
- Honduras
- Hong Kong
- Hungary
- Iceland
- India
- Indonesia
- Iran
- Iraq
- Ireland
- Isle of Man
- Israel
- Italy
- Jamaica
- Jan Mayen
- Japan
- Jersey
- Jordan
- Juan de Nova Island
- Kazakhstan
- Kenya

- Kiribati
- Korea, North
- Korea, South
- Kuwait
- Kyrgyzstan
- Laos
- Latvia
- Lebanon
- Lesotho
- Liberia
- Libya
- Liechtenstein
- Lithuania
- Luxembourg
- Macau
- Macedonia
- Madagascar
- Malawi
- Malaysia
- Maldives
- Mali
- Malta
- Marshall Islands
- Martinique
- Mauritania
- Mauritius
- Mayotte
- Mexico
- Micronesia, Federated States of
- Moldova
- Monaco
- Mongolia
- Montenegro
- Montserrat
- Morocco
- Mozambique
- Namibia
- Nauru
- Navassa Island
- Nepal
- Netherlands
- Netherlands Antilles

- New Caledonia
- New Zealand
- Nicaragua
- Niger
- Nigeria
- Niue
- Norfolk Island
- Northern Mariana Islands
- Norway
- Oman
- Pakistan
- Palau
- Panama
- Papua New Guinea
- Paracel Islands
- Paraguay
- Peru
- Philippines
- Pitcairn Islands
- Poland
- Portugal
- Puerto Rico
- Qatar
- Reunion
- Romania
- Russia
- Rwanda
- Saint Helena
- Saint Kitts and Nevis
- Saint Lucia
- Saint Pierre and Miquelon
- Saint Vincent and the Grenadines
- Samoa
- San Marino
- Sao Tome and Principe
- Saudi Arabia
- Senegal
- Serbia
- Seychelles
- Sierra Leone
- Singapore
- Slovakia
- Slovenia

- Solomon Islands
- Somalia
- South Africa
- South Georgia and the South Sandwich Islands
- Spain
- Spratly Islands
- Sri Lanka
- Sudan
- Suriname
- Svalbard
- Swaziland
- Sweden
- Switzerland
- Syria
- Taiwan
- Tajikistan
- Tanzania
- Thailand
- Timor-Leste
- Togo
- Tokelau
- Tonga
- Trinidad and Tobago
- Tromelin Island
- Tunisia
- Turkey
- Turkmenistan
- Turks and Caicos Islands
- Tuvalu
- Uganda
- Ukraine
- United Arab Emirates
- United Kingdom
- United States
- Uruguay
- Uzbekistan
- Vanuatu
- Venezuela
- Vietnam
- Virgin Islands
- Wake Island
- Wallis and Futuna

- West Bank
- Western Sahara
- Yemen
- Zambia
- Zimbabwe

3. **What is your gender?**
Mark only one oval.
- Female
- Male
- Prefer not to say
- Other: ____________________

4. **Are you part of the pet animal industry?**
Mark only one oval.
- No. Other profession not related to the animal industry
- Veterinarian or Vet Nurse
- Student of veterinary medicine, veterinary science or veterinary nursing
- Breeder
- Animal Services (groomer, farrier, animal acupuncturist, kennel staff, etc.)
- Animal Industry (pet food, pet toys, pet products)

5. **Pick your age group.**
Mark only one oval.
- 18-24
- 25-35
- 36-45
- 46-55
- 56-65
- Over 65

6. **What is your approximate annual household income? (Please include currency and amount)**

____

7. **What are your personal dietary preferences?**
Mark only one oval.
- Omnivore (meat and plant based diet)
- Vegetarian
- Vegan
- Other: ____________________

8. **Are there children under 10 years of age in your household?**
Mark only one oval.
- Yes
- No

9. **Are you or someone in your household immunocompromised?**
Mark only one oval.
- Yes
- No
- I don't know

10. **Are you or someone in your household pregnant?**
Mark only one oval.
- Yes
- No
- I don't know

11. **Have you spoken about your dog's nutrition with your veterinarian?**
Mark only one oval.
- Yes
- No

12. **On a scale from 1 to 5, how knowledgeable do you feel your veterinarian is about animal nutrition?**
Mark only one oval.

	1	2	3	4	5	
Least						Most

13. **On a scale from 1 to 5, how knowledgeable do YOU feel about your dog's nutrition?**
Mark only one oval.

	1	2	3	4	5	
Least						Most

About your dog's diet

Please answer the following questions about your dog(s).

14. **What does your dog eat most of the time (their main diet)?**
Mark only one oval.
- Commercial dry/kibble or wet/canned/tinned/sachet
- Prescription diet for a medical condition
- Commercially prepared raw food
- Homemade cooked diet
- Homemade raw diet

15. **How did you establish your dog's current diet?**
Mark only one oval.

- Recommendation of a veterinarian or vet nurse/technician
- Recommendation of the breeder or rescue/shelter
- Recommendation in breed specific literature
- Recommendation of friends or family
- Information published by a veterinarian or veterinary nutritionist
- Information published online from a non-veterinary source
- Tradition (always what you have fed)
- Other: ______________________

16. **What specifically made you choose that diet? Rank in order of importance where 1 is the most important and 8 is the least important.**
Mark only one oval per row.

	1	2	3	4	5	6	7	8
Ingredients								
Recommendation								
Convenience								
Environmental factors (sustainability)								
Nutrients								
Health of my dog								
Cost								
How much my dog likes the food								

17. **Does your vet approve of your dog's diet?**
Mark only one oval.

- Yes
- No
- I don't know

18. **Has your veterinarian ever discussed raw diets with you?**
Mark only one oval.

- Yes
- No

19. **What benefits are you aware of associated with feeding a raw diet?**

____

____

____

____

____

20. **What risks are you aware of associated with feeding a raw diet?**

Commercial Raw Diets

Please answer the following questions about commercially prepared raw diets.

21. **On a scale of 1 to 5, where do you rank raw commercial diets in terms of nutrition?**
Mark only one oval.

	1	2	3	4	5	
Least nutritious	◯	◯	◯	◯	◯	Most nutritious

22. **On a scale of 1 to 5, where do you rank raw commercial diets in terms of risk to human health?**
Mark only one oval.

	1	2	3	4	5	
Least risk	◯	◯	◯	◯	◯	Most risk

23. **On a scale of 1 to 5, where do you rank raw commercial diets in terms of risk to dog health?**
Mark only one oval.

	1	2	3	4	5	
Least risk	◯	◯	◯	◯	◯	Most risk

Homemade Raw Diets

Please answer the following questions about homemade raw diets.

24. **On a scale of 1 to 5, where do you rank homemade raw diets in terms of nutrition?**
Mark only one oval.

	1	2	3	4	5	
Least nutritious	◯	◯	◯	◯	◯	Most nutritious

25. **On a scale of 1 to 5, where do you rank homemade raw diets in terms of risk to human health?**
Mark only one oval.

	1	2	3	4	5	
Least risk	◯	◯	◯	◯	◯	Most risk

26. **On a scale of 1 to 5, where do you rank homemade raw diets in terms of risk to dog health?**
Mark only one oval.

	1	2	3	4	5	
Least risk	◯	◯	◯	◯	◯	Most risk

Commercial Dry or Wet

Please answer the following questions about commercially prepared dry or wet diets.

27. **On a scale of 1 to 5, where do you rank commercially prepared dry or wet diets in terms of nutrition?**
Mark only one oval.

	1	2	3	4	5	
Least nutritious	◯	◯	◯	◯	◯	Most nutritious

28. **On a scale of 1 to 5, where do you rank commercially prepared dry or wet diets in terms of risk to human health?**
Mark only one oval.

	1	2	3	4	5	
Least risk	◯	◯	◯	◯	◯	Most risk

29. **On a scale of 1 to 5, where do you rank commercially prepared dry or wet diets in terms of risk to dog health?**
Mark only one oval.

	1	2	3	4	5	
Least risk	◯	◯	◯	◯	◯	Most risk

30. **Select all the statements that you believe to be true.**
Check all that apply.

- [ ] My dog has the same nutritional needs as a wolf
- [ ] My dog's body has not evolved to digest carbohydrates
- [ ] My dog is a carnivore
- [ ] Processed food is unhealthy
- [ ] Dogs on a raw diet live longer
- [ ] None of these statements are true

Please write NA if any of the following questions do not apply to you.

31. **If you feed your dog a raw diet, why?**

____
____
____
____
____

32. **If you do not feed your dog a raw diet, why not?**

____

____

____

____

____

33. **If you don't feed a raw diet to your dog, have you ever considered it?**

Mark only one oval.

- ( ) Yes
- ( ) No
- ( ) Not applicable; I feed raw.

34. **Why or why not?**

____

____

____

____

____

Thank you for completing this survey!

Powered by Google Forms
